# Inteins and affinity resin substitutes for protein purification and scale up

**DOI:** 10.1186/1475-2859-4-32

**Published:** 2005-11-11

**Authors:** Mahmoud Reza Banki, David W Wood

**Affiliations:** 1 Department of Chemical Engineering, A213 E-QUAD, Princeton University, Princeton, NJ 08544, USA

## Abstract

The development of self-cleaving fusion-tag technology has greatly simplified the purification of recombinant proteins at laboratory scale. The self-cleaving capability of these tags has recently been combined with additional purification tags to generate novel and convenient protein purification methods at a variety of scales. In this review, we describe some of these methods, and provide a rudimentary economic analysis of hypothetical large-scale applications. This work is expected to provide a rough outline for the evaluation of these methods for large-scale bioprocessing of a variety of products.

## Introduction

An important development in the area of recombinant protein purification has been the incorporation of self-cleaving protein elements into a variety of fusion-based purification systems [1-3]. These elements are derived from naturally occurring self-splicing inteins through various protein engineering strategies, and have been combined with conventional affinity tags in a variety of configurations to yield highly effective separations methods. Very recently, these elements have also been combined with non-conventional purification tags to yield "self-purifying" proteins, which can deliver highly purified native products using simple mechanical means without chromatographic methods [4,5].

This review will compare conventional affinity-tag methods, with and without proteolytic tag removal, to three newer methods based on self-cleaving purification tags. The three newer methods include a conventional affinity tag separation with a self-cleaving chitin-binding tag (the IMPACT system), a more recent method where the expression host produces a granular affinity matrix during fermentation (the PHB system), and a third in which the target protein is tagged with a reversibly-precipitating self-cleaving polypeptide (the ELP system). In particular, the advantages and disadvantages of each method will be compared, and the large-scale economics of each of these systems will be examined from a simple raw-materials cost standpoint. This simple analysis is intended to describe the relative merits of these methods, and to provide an initial benchmark for evaluating their potential future role in the large-scale manufacture of recombinant products.

## Conventional Affinity-Tag Methods

Affinity fusion-based protein purification is a simple and now widely used method which takes advantage of the selective binding property of a genetically fused binding protein (tag) to purify a given target protein [6,7]. In place of physicochemical properties of the target protein, this technique relies on the specific binding of the affinity tag to an immobilized ligand. By exploiting this highly specific interaction, a single purification step can effectively isolate and purify a given target protein with ease. The development of numerous tags has further demonstrated the flexibility and potential of this method. Despite these strengths, however, the use of conventional gene-fusion affinity tags suffers from two main drawbacks.

The first limitation arises from the requirement that the tag be removed in order to recover a native target protein. This is generally accomplished by enzymatically removing the tag from the purified target by the addition of an appropriate protease. To facilitate this procedure, the target sequence of the selected protease is genetically included between the tag and the target protein when the fusion is constructed, allowing specific cleaving to take place. Although this procedure is generally effective at laboratory scales, the cost of protease enzymes is prohibitive at manufacturing scale. In addition, yield losses can arise from incomplete cleaving or unexpected cleaving within the target, and the affinity tag and protease must also be separated from the cleaved target protein in a separate purification step. Both of these aspects increase the cost and complexity of the purification, while decreasing the yield.

A second limitation arises from the equipment and consumable resin costs associated with these procedures. Conventional affinity resins typically consist of various cross-linked polymers, derivitized with appropriate ligands at the end of optimized spacer arms. Manufacturing costs for these resins are typically much higher than for ion-exchange and other chromatography resins, which can offset the appeal of the simpler affinity-based separation. A notable exception has been the widespread use of Protein A affinity columns in the purification of antibody therapeutics. However, this separation is limited to native antibodies, without the addition of a fusion tag. This suggests that conventional affinity tag methods may be attractive if tag removal can be simplified [8].

For these reasons, new methods which eliminate the need for protease treatment and expensive affinity resins are likely to make a significant impact on large-scale protein purification processes or high-throughput screening of protein libraries. The next two sections address the two drawbacks mentioned above and offer recently developed solutions.

## Self-cleaving Affinity Tags

Inteins (INTervening protEINS) are naturally occurring protein sequences capable of post-translational self-excision from a host-intein precursor protein through a process known as "protein splicing" [9,10]. Several intein examples have been identified where the intein is capable of functioning outside of its native context, allowing these inteins to be developed for a variety of biotechnological applications. One of the most significant of these is the creation of self-cleaving protein elements that can be combined with conventional affinity tags to generate effective self-cleaving affinity tags [1-3]. A critical feature of these tags is their ability to release a target protein, fused either C or N-terminally to the tag, in response to a simple chemical or physical stimulus. The highly specific cleaving reaction thus allows the affinity tag to be removed without the addition of expensive protease, while at the same time preventing unwanted cleaving. An additional important advantage is that the cleaving reaction can be induced while the tagged target is bound to the affinity column, thus eliminating the need for subsequent removal of the cleaved tag.

The first commercially available intein purification system was developed by New England Biolabs (NEB), and is based on a modified *Saccharomyces cerevisiae *vacuolar ATPase subunit A intein (*Sce *VMA intein) [1]. This intein possesses a particular mutation, leading it to exhibit N-terminal cleaving in the presence of 30 mM 1,4-dithiothreitol (DTT) or β-mercaptoethanol over a wide pH range (5.5 – 9.0). This intein was combined with a chitin binding domain and appropriate resin to form the IMPACT system, and has been effective in the purification of several proteins and restriction enzymes at laboratory scale. The original IMPACT system has now been enhanced to form the IMPACT-CN system, where C-terminal cleaving can also be induced through the addition of DTT to an appropriate fusion precursor. Thus both N and C-terminally fused target proteins can be purified, allowing the design of the fusion to be optimized for expression and folding of the target. NEB has more recently expanded their line of intein-based separation systems to include three modified mini-inteins derived from the *Mycobacterium xenopi *GyrA enzyme, the *Synechocystis spp*. strain PCC6803 DnaB helicase, and the *Methanothermobacter thermautotrophicus *Ribonucleoside-diphosphate reductase enzyme [2,11,12]. These inteins are included in the pTWIN system, and provide the capability to induce cleaving by thiol addition (such as DTT or β-mercaptoethanol as above), small shifts in pH or increases in temperature.

In addition to these commercially available inteins, an engineered mini-intein derived from the *Mycobacterium tuberculosis *(Mtu) RecA intein has been developed independently [3,13]. This 18 kDa intein was developed through a deletion of the endonuclease domain from the native Mtu RecA intein, followed by mutagenesis and selection for rapid and controllable cleaving. This intein, referred to as the ΔI-CM mini-intein, can be controlled by pH and temperature to yield isolated C-terminal cleavage, and has been successful in delivering native, purified target proteins from various *E. coli *expression systems. It is this intein that has been combined with the novel purification tags described above to generate convenient "self-purifying" expression systems. The potential of these systems in the purification of recombinant proteins at large scale will be examined in the following sections.

## The PHB System

Polyhydroxybuterates (PHBs) are a subclass of biodegradable polymers produced in various organisms and are generally thought to be a means for storing excess carbon in the absence of oxygen, nitrogen or phosphorus [14]. Intracellular PHB takes the form of small granules when expressed, which can vary in morphology based on the expressing organism, the carbon source, and the expression level of accompanying proteins called phasins [15]. The specific affinity of phasin proteins for PHB has been exploited in the development of a self-contained affinity purification system [5]. In this case, the expressing cells harbor two plasmids; one expresses the PHB-synthesis genes, while the other expresses a target protein in fusion to a self-cleaving phasin tag. The large molecular weight of PHB granules allows the simple recovery and cleaning of the granules and bound fusion protein, while the self-cleaving intein allows the purified native target protein to be released from the granule surface once it is purified. Because PHB granules can be readily synthesized from cheap carbon sources such as glucose or lactate, this system provides a low-cost alternative to manufactured and processed affinity beads. Utility of PHB granules in purification has been described, where a phasin tag has been combined with the ΔI-CM mini-intein and used for the purification of several active proteins with competitive yields [5].

## The ELP System

Another alternative to conventional resins is a recombinantly produced elastin-like polypeptide (ELP), generally comprised of repeating units of the five amino acids VPGXG (X = any amino acid) [16,17]. Because of the unique salt and temperature-sensitive solubility of ELP, it can be easily purified by salt addition and mild temperature shifts. By combining an ELP tag with the ΔI-CM mini-intein, a method has been created which allows the rapid and simple purification of arbitrary tagged target proteins [4]. In this case, the tagged target is separated from the insoluble components of the cell debris by centrifugation at low temperature, where the ELP is soluble. Addition of salt and an increase in temperature to 30°C causes the ELP portion of the fusion to self-assemble into an insoluble precipitate, allowing it to be easily separated from the remaining soluble components of the cell lysate. Because the precipitation is limited to the ELP portion of the protein, which is separated from the intein and target by a flexible linker peptide, the activity of the intein and target are not affected. Intein cleaving then releases the native target from the ELP tag, which can then be easily separated by an additional precipitation step. This method is compatible with both centrifugation and filtration for recovering and separating the ELP-fusion precipitate. Initial reports indicate that this technique is highly effective in purifying active and native protein products expressed in *E. coli*, although it is anticipated it will be compatible with a large number of expression systems.

## Large-Scale Economics

**Figure 1:**
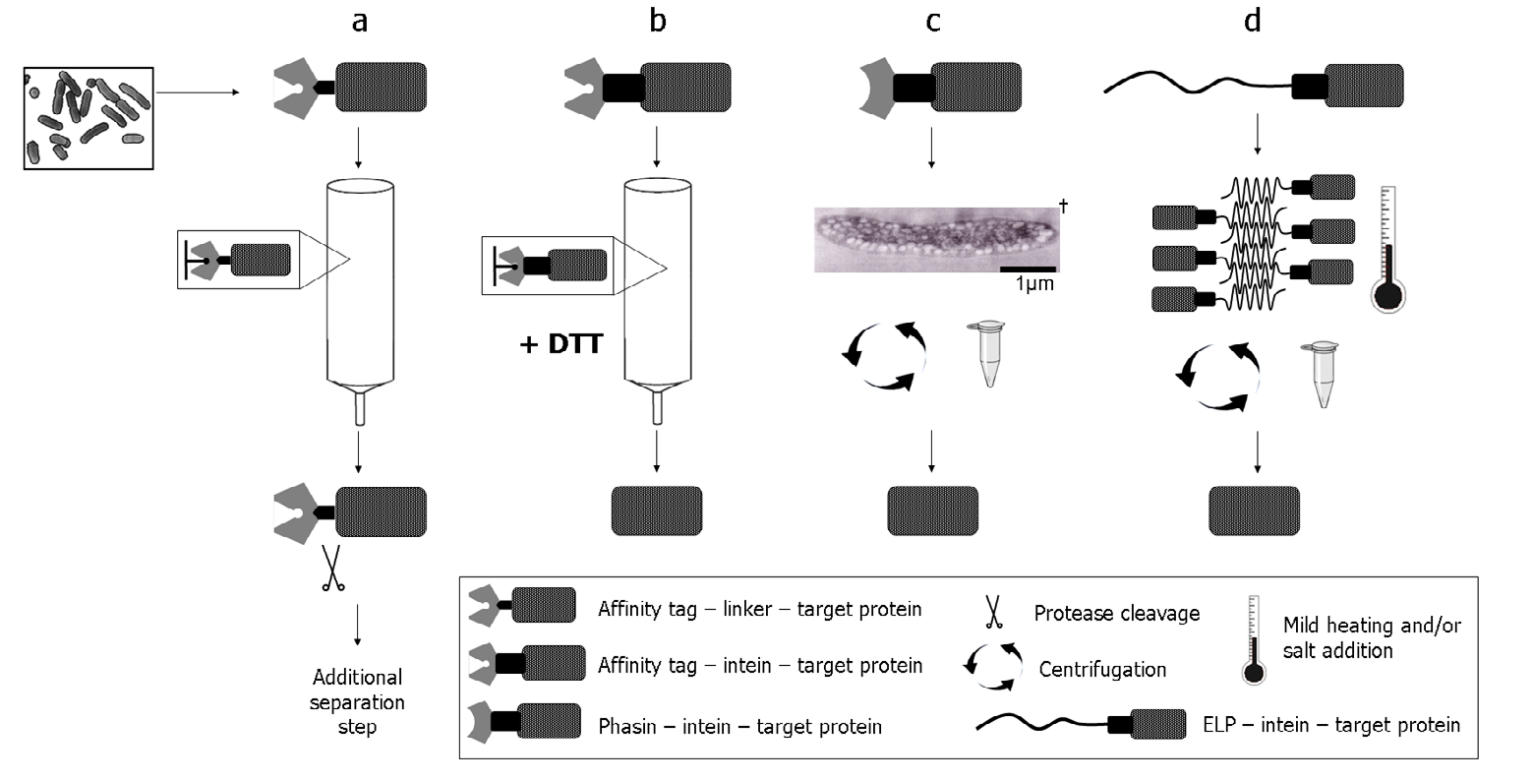
Protein purification schematic for (a) conventional affinity-base purification (pMAL), (b) intein-based affinity purification where the linker between the affinity tag and the target protein is replaced by a self-cleaving intein, (c) PHB-intein mediated protein purification where *in vivo *PHB granules substitute for the affinity resin, and (d) ELP-intein mediated protein purification where an *in vivo *temperature sensitive, self-cleaving tag replaces both the affinity resin and the affinity tag. † Scanning electron micrograph image of PHB granules expression in *E. coli *was taken from Reference [20].

**Figure 2:**
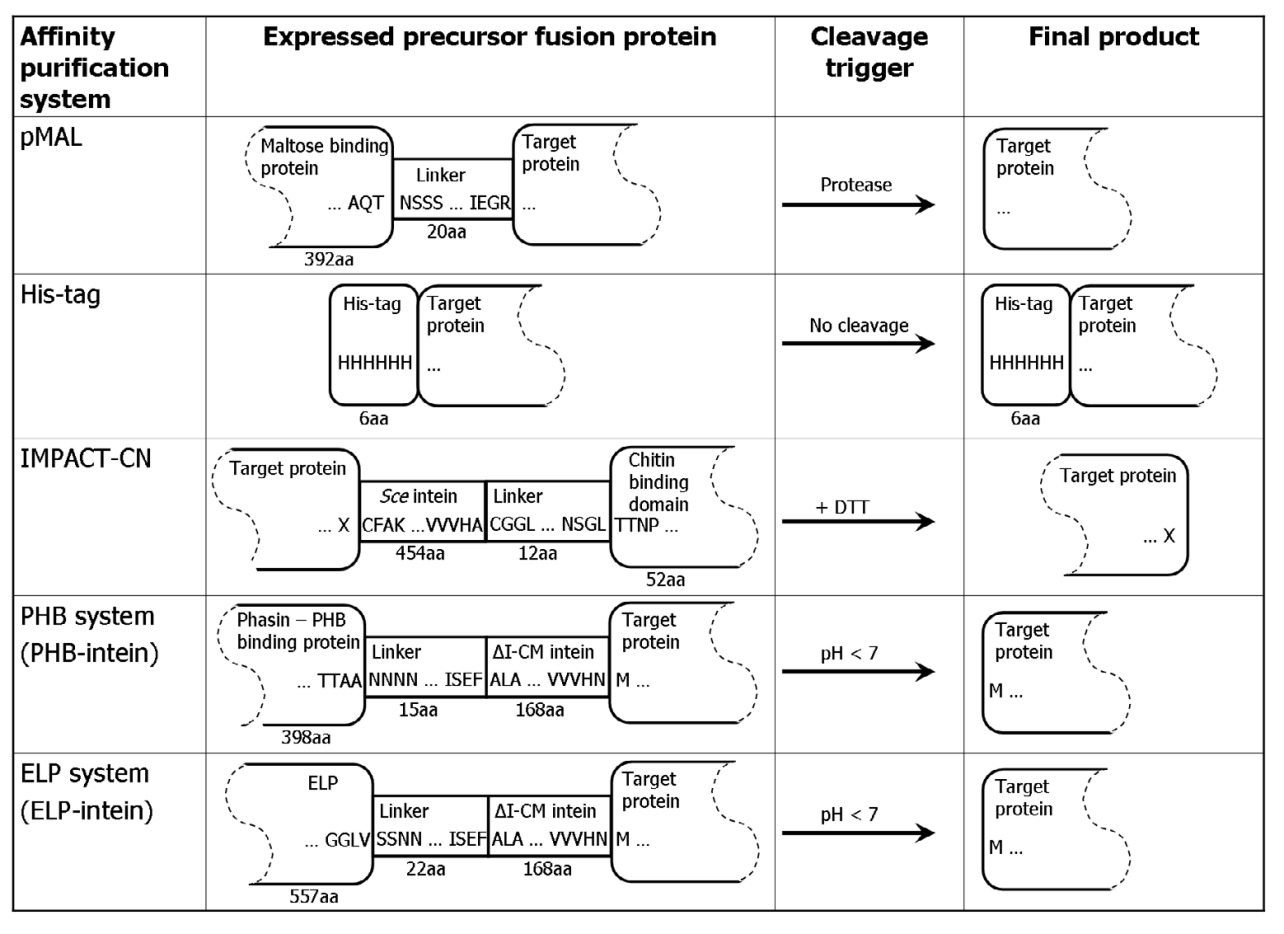
Comparison of triggers of cleavage as well as final products for the five methods. Amino acid sequences at fusion junctions are noted in one-letter amino acid code. X denotes that specific amino acids are preferred for this position.

Despite the clear potential of intein-based separations in industry, little has been done to adapt these methods to large-scale processes. Recent work describes the activity of inteins expressed in high cell-density fermentation, and one recent paper examines the use of inteins with vortex-flow affinity-resin loading [18,19]. Here, we present a comparison of several conventional and intein-based affinity processes from a materials standpoint. Two conventional affinity-based purification methods were used as benchmarks: the maltose-binding protein fusion with proteolytic tag removal (pMAL) and His-tagged purification without tag removal. The pMAL system is available from New England Biolabs (Beverly, MA, USA) and we have chosen the Novagen (Madison, WI, USA) His-bind Purification System from a number of available His-tag purification systems. These two techniques are frequently used for small, lab-scale processes. Despite the high purities attainable with these two systems, they have not yet been adopted for large-scale enzyme production primarily due to the high cost of proteases and affinity resins. The IMPACT system, which circumvents the protease problem through DTT-induced intein cleaving, is the third system we have analyzed. Finally, two recently-developed PHB and ELP methods, which allow pH-induced intein cleaving by the ΔI-CM mini intein and virtually eliminate affinity resin costs, are also included for comparison (Figure 1). Comparison of triggers of target protein cleavage and recovery from the precursor protein fusions are also shown for all five methods (Figure 2).

A materials-based cost comparison can be a decisive prelude to the adoption of the PHB-intein and ELP-intein methods and also in predicting the significant cost savings possible with these two new technologies. Our economic analysis (Table 1) is based on published technical manuals from New England Biolabs (for the Maltose-binding and IMPACT methods), Novagen (for the His-tag method) and published work (for the PHB and ELP methods), and is limited to the consumable costs associated with each process. For this analysis, prices were calculated using supplier list prices for the largest quantities available (best rates) and are at times extrapolations on small-scale amounts. Although bulk order of chemicals and materials could further reduce the cost, the comparison presented here uses the same cost basis for all five methods and hence does not favor a method over another in individual sub-categories. Furthermore, costs associated with pH adjustments, ultrapure water, centrifugation, cooling, heating and plant operation are not considered due to their commonality in all of the processes. Finally, the induction (IPTG) cost is listed separately because it can potentially be eliminated in all five methods by using self-inducing expression strains, some of which are now commercially available (Novagen).

The pMAL and His-tag methods have been commercialized and have thus matured and been optimized for buffer consumption. Therefore, these two methods are most sensitive to the resin cost; a cost that can not be reduced or compromised. However, in the newly developed intein-dependent methods, the growth media and buffers are a larger fraction of the total cost. These components can potentially be replaced by cheaper alternatives, adding to the economic attractiveness of the PHB and ELP methods.

Even without exhaustive buffer and growth medium optimization, this comparison shows up to a 125 fold decrease in materials cost for the PHB and ELP methods in comparison to the pMAL affinity based purification. Likewise, these two technologies reduce the materials cost up to 11 fold in comparison with the His-bind purification procedure. This is a dramatic improvement over previously existing technologies and could thus have a significant impact on the future of the biotechnology industry.

## Conclusion

The cost analysis presented here shows the dramatic improvements possible for large-scale protein purification processes through the use of non-chromatographic self-cleaving purification tags. These methods are immediately attractive for large-scale industrial products, where small levels of impurities are tolerable. In pharmaceutical and other applications where high purity is required, these methods can act as a first-capture step, delivering substantially purified material for downstream polishing. In addition, these methods are ideal for high throughput applications, where the simplicity and generality of each method can be applied to large libraries of targets in a highly parallel configuration. As the biochemistry associated with self-cleaving tags is further optimized, it is clear that this platform will be adaptable to many additional separation processes.

## Figures and Tables

**Table 1:** Material cost comparison for five different protein purification methods based on 1 kg product protein yield†

	**pMAL fusion w/tag removal**	**His-tag w/o tag removal**	**IMPACT-CN**	**PHB System**	**ELP System**
**Supplier**	**NEB**	**Novagen**	**NEB**	**WOOD LAB**	**WOOD LAB**

**Typical yield**	40 mg/L	74 mg/L	4.5 mg/L	36 mg/L	85 mg/L
**Resin capacity**	3 mg/ml	8 mg/ml	2 mg/ml	-	-
**Growth medium**	$ 44,198.38	$ 23,101.67	$ 379,894.17	$ 126,257.54	$ 58,699.27
**Induction cost**	$ 35,373.35	$ 19,101.61	$ 314,115.30	$ 39,304.03	-
**Buffer cost**	$ 51,900.11	$ 5,921.45	$ 103,596.75	$ 27,212.63	$ 15,810.47
**Resin cost**	$ 624,375.00	$ 790,000.00	$ 192,000.00	-	-
**Denaturation cost**	$ 428,835.99	-	-	-	-
**Protease cost**	$ 8,480,000.00	-	-	-	-
**Total cost per kg of product**	$ 9,664,682.82	$ 838,124.73	$ 989,606.23	$ 153,470.17	$ 74,509.75

† Cost calculations are based on a simple scaling-up of published protocols for laboratory-scale processes. Bulk prices of chemicals and growth media components were obtained from the best available rates per unit mass from Fisher Scientific. Items specific to individual methods were priced by the supplier (i.e. the protease cost was obtained from NEB, His-tag resin cost was obtained from Novagen, etc). Required quantities of all components (i.e. growth media, buffers, resin) were calculated based on the typical yields of each process for a final product yield of 1 kg. In addition, the following assumptions are included for each method: pMAL method – Yield estimate and material usage requirements were based on the supplier, NEB, recommendations in the pMAL Protein Fusion and Purification System manual [21]. In place of the additional DEAE sepharose ion exchange chromatography step for separating the protease and maltose tag, it was assumed that the same amylose resin would be used twice with regeneration. Therefore, one round of purification uses the amylose resin twice. Therefore with an assumed regeneration of four times, the same bed can be used for two separate purifications. Amylose resin binding capacity is 3 mg of fusion protein per ml of resin. This translates to 1 to 2 mg of target protein per ml of resin depending on the molecular weight of the target protein. Even so, it was assumed that 3 mg of maltose tag binds to each ml of resin, hence, underestimating the amylose resin cost. The recommended amount of protease (Factor Xa) is 1% (w/w) of the fusion protein, hence, if an average product protein 1/3 the size of the maltose binding domain tag is used, 4000 g of fusion is needed to produce 1000 g of target protein assuming perfect recovery. Despite this recommendation, the quantity of protease used was based on 1000 kg of fusion protein as opposed to target protein, hence, underestimating the protease cost. His-tag method – Typical yield was based on the published yield using Novagen Standard HisBind for purification of HisTag GST expressed in *E. coli *[22]. Material requirements were based on supplier (Novagen) recommendations for the HisBind kit [23]. In these calculations the protease step is not factored into the cost and the target protein retains the HisTag after purification. The regeneration is not taken into account because Novagen recommends using a different resin for each different protein. However, a routine regeneration single-step procedure or complete resin regeneration (16 steps) are available even though not considered here. IMPACT-CN method – The yield estimate was based on the average yield published by the supplier, NEB, in the IMPACT-CN manual [24]. Purified protein examples included in this estimate were [24]: Maltose-binding protein, McrB, T4 DNA ligase, Bst DNA polymerase large fragment, BamH I, Bgl II, CDK2, CamK II, T4 Gene 32 product, FseI GFP, CamK II, Invertase, and T4 Endo VII. It was assumed that the chitin beads can be regenerated 5 times as recommended by the manual. PHB method – Excess DTT in buffers for this method is only necessary for specific target proteins and has not been factored into the cost. In addition, cheaper phosphate substitute buffer is used in place of Bis-Tris in the calculation. IPTG induction does not significantly change the yield and has not been used in the calculation. Cost of purification for this method prior to these three modifications (DTT and IPTG elimination as well as Phosphate for Bis-Tris substitution) is $391,265.84 as opposed to the listed $153,470.17. ELP method – This method does not involve sonication and cell lysis can be achieved with 0.2 mg/ml lysozyme content in the lysis buffer (unpublished data) as opposed to the 1 mg/ml published [4]. Furthermore, glycerol was not included in the TB media as noted in the publication. In addition cheaper phosphate buffer is substituted for the published Bis-Tris buffer in this calculation. Cost of purification for this method without this modification (Phosphate for Bis-Tris substitution) is $122,754.12 as opposed to the listed $74,509.75.
